# Source, Monitoring Techniques and Prospects of Bioaerosols: A Review

**DOI:** 10.3390/toxics14050404

**Published:** 2026-05-08

**Authors:** Jiaqing Wu, Chun Chen, Hong Geng, Bixin Zhao, Jian Gao

**Affiliations:** 1 State Key Laboratory of Environmental Criteria and Risk Assessment, Chinese Research Academy of Environmental Sciences, Beijing 100012, China; wujiaqing@sxu.edu.cn; 2 Institute of Environmental Science, Shanxi University, Taiyuan 030006, China; genghong@sxu.edu.cn (H.G.); zhaobixin@sxu.edu.cn (B.Z.)

**Keywords:** bioaerosols, sources, monitoring methods

## Abstract

Bioaerosols play significant roles in ecological interactions, climate change, and public health. Their diverse origins contribute to a dynamic atmospheric microbiome with considerable spatiotemporal variability, which are generally categorized as natural and anthropogenic sources. Accurate monitoring and source apportionment are critical for assessing environmental impacts and health risks. This review systematically summarizes the characteristics of bioaerosol sources and emphasizes emission risks from intensive human activities. This study also elucidates source apportionment strategies of bioaerosols and analyzes the technological evolution from traditional culture-based methods to advanced molecular and real-time physicochemical systems. In addition, the shift of bioaerosol monitoring technologies towards high-sensitivity, culture-independent, and online monitoring is emphasized in this review. An outlook on future research priorities is provided in the end. We emphasize the pressing need to establish localized characteristic databases, develop integrated real-time monitoring systems coupling rapid screening with deep biological analysis, and optimize the application of machine learning and AI algorithms to enhance the precision of multi-source contribution modeling in complex environments.

## 1. Introduction

Bioaerosols, defined as biological particulate matter suspended in the atmosphere, encompass a broad spectrum of particle sizes ranging from nanometer-scale viruses to millimeter-scale pollen grains [1]. They are composed of diverse constituents, including bacteria, fungi, viruses, archaea, pollen, plant debris, as well as biological macromolecules such as endotoxins and proteins [2]. Bioaerosols construct a complex interaction network among Earth’s biosphere, atmosphere, and human health. Consequently, their environmental dynamics and associated health effects have consequently emerged as a prominent research focus within contemporary environmental science and public health disciplines. In addition, certain bioaerosols can serve as cloud condensation nuclei (CCN) or ice nucleating particles (INP), directly participating in cloud formation and precipitation processes [3]. These roles enable them to affect hydrological cycles and climate patterns across regional and global scales. Furthermore, bioaerosols act as transmission vectors for various pathogenic microorganisms, allergens, and toxins. Prolonged or acute exposure to high concentrations of bioaerosols has been associated with respiratory conditions such as asthma, allergic rhinitis, chronic obstructive pulmonary disease (COPD), and various respiratory infectious diseases [4,5].

Given the diverse origins and complex composition of bioaerosols, establishing a precise and efficient monitoring and source tracking system is critical. For an extended period, bioaerosol monitoring has primarily depended on culture-based sampling techniques such as the Andersen impactor [6]. However, traditional culture methods are time-consuming and fail to detect the substantial proportion of viable but non-culturable (VBNC) microorganisms in the environment, frequently leading to an underestimation of actual bioaerosol concentrations [7]. In recent years, with the rapid development of molecular biology and optoelectronic detection technologies, culture-independent techniques represented by high-throughput sequencing and quantitative PCR (qPCR), as well as real-time online monitoring methods based on laser-induced fluorescence and mass spectrometry, have progressively emerged as mainstream research tools [7,8].

This study systematically reviews the characteristics of major bioaerosol sources and analyzes their potential impacts on the environment and health. In addition, the evolutionary history and latest progress of bioaerosol monitoring technologies are outlined, as well as the advantages, disadvantages, and applicable scenarios of mainstream detection technology. Building upon the current state of development in bioaerosol source tracking and detection technologies, this paper further proposes scientifically grounded directions for future research in the field.

## 2. Sources of Bioaerosols

**Figure 1:**
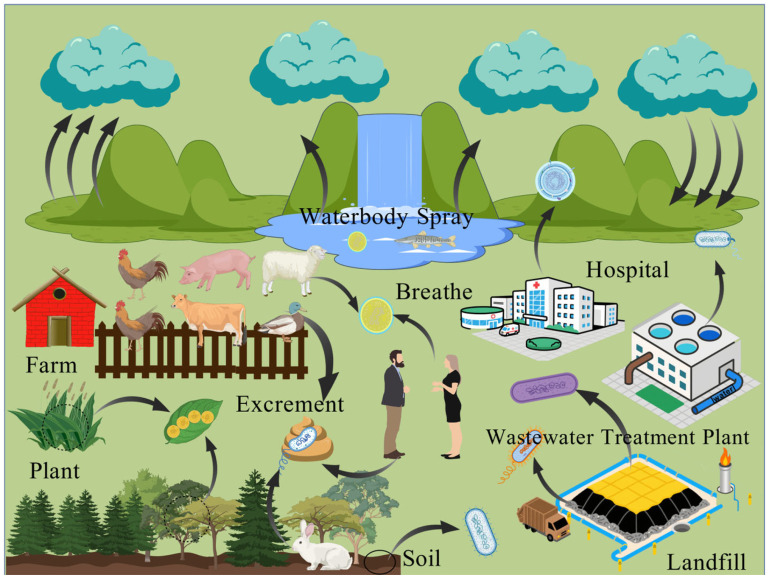
Sources of bioaerosols (Created with BioGDP.com [9]).

Bioaerosols originate from a wide range of sources, which can be categorized into natural sources, (e.g., plant, soil, waterbody) and anthropogenic sources (i.e., various man-made systems), as shown in Figure 1. The contribution of these sources to atmospheric bioaerosol composition exhibits significant spatiotemporal variation, which effectively mirrors the migration and diffusion patterns of microorganisms within the ecosystem.

### 2.1. Natural Sources

(1) Plants: Plant leaves are an active natural source of atmospheric bioaerosols and a major global habitat for microorganisms (including bacteria, fungi, viruses, cyanobacteria, actinomycetes, nematodes, and protozoa) globally [10]. The abundance and species of bacteria and fungi in the atmosphere of urban and rural areas are correlated with the extent and type of urban green spaces [11,12]. Plant-mediated emissions of bioaerosols exhibit pronounced seasonal dynamics. Previous studies indicate that the abundance of taxa closely related to the phyllosphere microenvironment increases significantly in summer atmospheric samples, such as *Pseudomonadaceae* and *Sphingobacteriaceae* [13]. *Sphingomonadales* as a leaf-enriched bacteria maintain a relatively high abundance in the summer atmosphere [14]. It is mainly attributed to the vigorous growth of plant leaves in warm seasons, which leads to more active associated microbial communities, thereby resulting in a sustained atmospheric release of characteristic bacterial groups.
(2) Soil: Soil serves as a prominent natural source of airborne microorganisms [15,16]. As a reservoir of immense microbial diversity, a single gram of soil can constitute an amount on the order of one million distinct microbial genomes [17]. In addition, microbial communities exhibit distinct compositional stratification at varying altitudes above the soil surface [18]. The decline in leaf-derived bioaerosol emissions during plant senescence corresponds to an increased relative contribution from soil sources to the atmospheric bioaerosol pool in autumn and winter. For instance, Bowers et al. [13] observed an increase in the relative abundance of *Bacillales* in autumn samples, which are considered a key indicator taxon for soil origins.
(3) Natural Water Bodies: Aquatic microorganisms can be entrained into the atmosphere via wind-driven processes and tidal action, subsequently undergoing long-range atmospheric transport. Aller et al. [19] indicated that the sea surface microlayer (SML) serves as a primary reservoir and critical interface for the transfer of aquatic microorganisms to the atmosphere. On the other hand, freshwater is also an important source of atmospheric bioaerosols. Through environmental source apportionment, Liao et al. [20] found that freshwater environments are the dominant contributors to airborne microorganisms in Xiamen, with freshwater sources accounting for 31.07% and 58.76% of bacteria and eukaryotic microorganisms, respectively, revealing the significant influence of freshwater environments on shaping atmospheric microbial communities.

### 2.2. Anthropogenic Sources

(1) Humans: Humans act as prominent emission sources of atmospheric bioaerosols, releasing a diverse array of microorganisms originating from the skin, respiratory tract, and gut into indoor and ambient air. Skin-associated bacteria, such as *Staphylococcus* and *Streptococcus* [21], are released into the atmosphere via the shedding of epidermal cells. Meanwhile, respiratory microbiota are expelled in liquid droplets through expiratory activities such as talking, coughing, and sneezing [22]. These droplets suspended in the atmosphere constitute an important component of bioaerosols, thereby establishing a direct transmission route for agents such as influenza and coronaviruses. A single cough can release thousands of microbe-containing droplets, while the number of droplets released by sneezing is even more massive. Simultaneously, gut microorganisms are aerosolized via flatulence or excretion [23]. These human-driven emissions not only reshape the structure and diversity of atmospheric microbial communities but also introduce pathogen-laden aerosols into the air.
(2) Man-made systems: Man-made systems serve as major anthropogenic sources of atmospheric bioaerosols, and the resulting bioaerosol pollution has emerged as a critical public health issue. As shown in Table 1, wastewater treatment plants (WWTPs), landfills, livestock farms, and hospitals represent significant anthropogenic sources of bioaerosols. Comparatively, the highest average concentrations of bacteria and fungi in microbial aerosols were determined in livestock farm, followed by WWTPs, landfills, and hospitals, sequentially.

During wastewater treatment, operations like agitation and aeration can disperse microorganisms from sewage into the atmosphere, forming bioaerosols [42]. Containing pathogenic agents and drug-resistant strains such as *Escherichia coli* and *Salmonella*, these bioaerosols present significant health hazards to employees at WWTPs [42]. Prolonged exposure may lead to a suite of respiratory conditions, including allergic rhinitis, asthma, chronic bronchitis, and hypersensitivity pneumonitis, which are referred to as Sewage Worker’s Syndrome. In addition, bioaerosols can also disperse into the surrounding environment, posing potential impacts on residents living nearby [43]. A previous study found that significant differences in the characteristics of bioaerosol characteristics vary among different treatment units in WWTPs. For instance, higher concentrations of bioaerosols were measured in grids and grit chambers, as well as sludge dewatering rooms, generally higher than those in aeration tanks, owing to poor ventilation conditions and insufficient air dilution [44]. Qiu et al. [45] indicated that the highest concentrations of bacterial and actinomycete aerosols were detected in the sludge dewatering workshop. Wang et al. [26] indicated that the biochemical reaction tanks exhibited the highest concentrations of bacterial aerosols. Notably, across various treatment units within the same WWTP, bacterial aerosol levels consistently exceeded those of fungal aerosols. This pattern can be attributed to the substantially higher bacterial content relative to fungal content in both sewage and sludge [46].

Excrement and secretions from livestock breeding are rich in microorganisms such as bacteria, viruses, and fungi. While animal coughs and sneezes can expel approximately 1 × 10^5^ biological droplets [47], animal manure remains the primary source of farm bioaerosols. For example, Duan et al. [48] demonstrated that *Escherichia coli* isolated from airborne samples in chicken coops showed 100% similarity with strains obtained from pig farm manure. Similarly, Chai et al. [49] pointed out that padding mixed with feces and urine serves as a major substrate for fungal proliferation. Under conditions of inadequate ventilation or poor management, these microorganisms can diffuse into the periphery of farms as bioaerosols via air currents, posing risks not only of triggering animal epidemics but also of threatening the health of nearby residents. Multiple studies have confirmed that endotoxins constitute a major component of bioaerosols around livestock farms [50,51,52,53]. Schinasi et al. [54] found that endotoxins could induce symptoms of acute respiratory infections among residents living within 1.5 miles of pig farms. The characteristics of farm bioaerosols are closely associated with the type of animals reared. In particular, poultry farms have been shown to produce significantly higher quantities of aerosols compared to other livestock operations. This is largely attributed to the incomplete digestive system of poultry, which results in undigested feed being excreted with manure. If not promptly removed, this accumulated waste generates substantial ammonia, creating favorable conditions for microbial growth [55,56]. Additionally, the flapping behavior of birds disturbs microorganisms settled on the ground, facilitating their entry into the air with dust particles. Other factors, including ventilation methods [57], stocking density [58], and the frequency environmental cleaning and disinfection [59], are key factors influencing the emission of airborne microorganisms within animal housing systems.

Landfills are a typical environment for the accumulation and proliferation of bioaerosols [60], with municipal solid waste generation and disposal processes constituting major sources and sinks of airborne microorganisms [61]. Landfills generally comprise landfill zones, cover zones, and leachate treatment facilities. Within the waste disposal zone, mechanical handling and turning operations release substantial amounts of microorganisms, forming microbial aerosols [62]. These aerosols often carry pathogens and antibiotic resistance genes (ARGs), posing considerable health risks to nearby residents and on-site workers. Inhalation exposure has been identified as the dominant risk pathway, with health risks estimated to be approximately four orders of magnitude higher than those associated with dermal contact [63]. Previous research indicated the positive correlation between PM_10_ concentration and culturable pathogen concentration downwind of landfills, suggesting a high respiratory exposure risk [64]. Growing attention is being directed toward the environmental dissemination of airborne ARGs in landfill settings. Yu et al. [65] demonstrated the horizontal transfer and environmental spread of antibiotic resistance via bioaerosols, where ARGs may achieve horizontal transfer through mobile genetic elements (MGEs), indicating that shifts in ARG profiles are largely driven by changes in bacterial host communities. Similarly, Morgado-Gamero et al. [66] also confirmed that bacterial bioaerosols in and around landfills exhibit resistance to multiple antibiotics. Substantial spatial variability in bioaerosol concentrations has been observed across different functional areas of landfills [67,68], with the highest concentration detected in active operational zones For example, Yang et al. [63] reported peak bioaerosol emissions in the working area, where the average airborne bacterial concentration reached 22,778 CFU/m^3^. Seasonal differences exist in landfill bioaerosol characteristics. Breza-Boruta [69] reported that microbial concentrations typically peak during the warmer spring and summer months, while the peak detected in autumn was determined by Yang et al. [63]. These discrepancies may reflect differences in site-specific operational practices, waste treatment processes, and local climatic conditions across landfill facilities.

### 2.3. Source Apportionment Methods

Bioaerosols serve as critical transmission vehicles for pathogens and allergens [70,71]. Accurate source apportionment is therefore imperative for mapping “source–concentration” dynamics and providing early warnings for susceptible populations. Currently, mainstream bioaerosol source apportionment approaches primarily encompass statistical and receptor modeling [72], database-driven functional mapping [73], and modeling methods [74].

(1) Correlation analysis is the most common and intuitive approach for bioaerosol source apportionment. The core principle involves determining similarity between samples via statistical measures to infer the potential sources of bioaerosols, such as Principal Component Analysis (PCA). However, correlation analysis generally requires large sample sizes and relatively high analytical costs. Meanwhile, it is not able to provide accurate quantitative estimates of contribution rates from individual sources [75]. Contemporary research has increasingly adopted advanced receptor models. Techniques such as Positive Matrix Factorization (PMF) [72] or co-occurrence network analysis [76] have now been deployed to integrate microbial community structures with specific chemical tracers. This integration overcomes the limitations of early statistical methods by enabling a more quantitative assessment of localized environmental drivers, although it still requires robust, long-term sampling datasets.
(2) Database-Driven Functional Mapping: Database analysis for bioaerosol source tracking relies on comparing characteristic signatures from known sources with those of unknown environmental samples. Widely used reference databases include BLAST [77], GCM [78], etc. While database-based analysis enables systematic comparison against established source profiles, it still faces several limitations. First, the biological properties of microorganisms are highly sensitive to local environmental conditions, which may differ substantially from those under which reference data were collected, leading to potential mismatches. Second, as the number of potential sources increases, the cost and complexity of database construction and maintenance rise significantly. To mitigate these limitations, recent methodological advances have increasingly focused on functional and ecological niche mapping. By converting taxonomic profiles into functional guilds or metabolic phenotypes [79], researchers are able to infer the original environmental habitats of bioaerosols, thus avoiding the prohibitive costs and regional biases associated with establishing comprehensive taxonomic reference databases.
(3) Model Analysis: In recent years, modeling has become an increasingly common approach for bioaerosol source apportionment. Early applications heavily relied on SourceTracker [80]. However, given that bioaerosols frequently contain a massive fraction of “unknown” environmental contributors, newer models like FEAST (Fast Expectation-Maximization for Microbial Source Tracking) have proven superior [81]. FEAST utilizes an expectation-maximization algorithm that significantly enhances computational efficiency and statistical accuracy when teasing apart weak source signals from dominating unknown backgrounds. Furthermore, to elucidate the mechanisms of long-range transport, local MST data are increasingly coupled with atmospheric dispersion models like HYSPLIT [82]. Advanced applications now integrate HYSPLIT with Potential Source Contribution Function (PSCF) [83] or Concentration Weighted Trajectory (CWT) [84] analyses, transforming qualitative air mass trajectories into quantitative spatial probability maps.

## 3. Progress in Monitoring Methods

**Figure 2:**
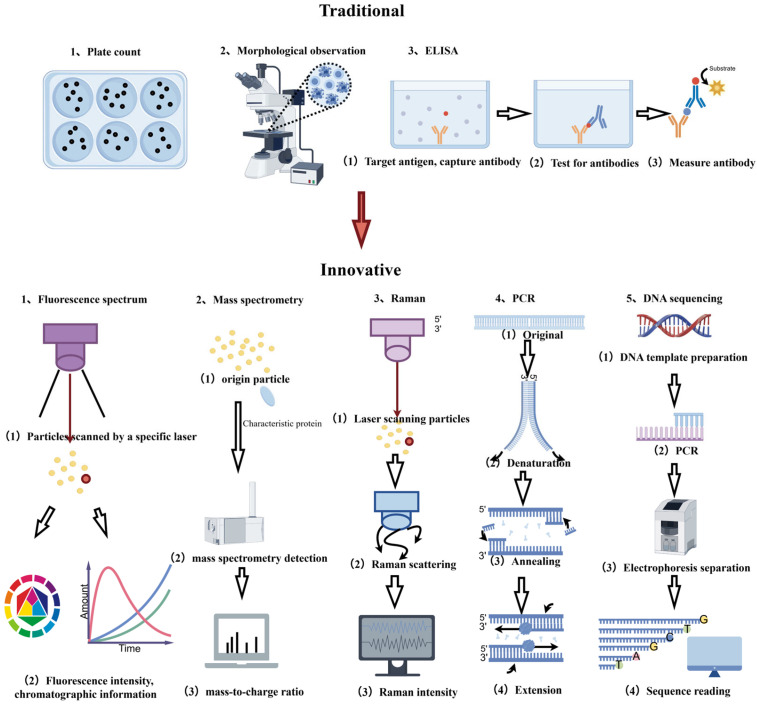
Bioaerosol detection methods (by Figdraw).

Bioaerosol monitoring encompasses a diverse range of methodologies, which can be broadly classified into six categories based on their detection principles and technical characteristics. As illustrated in Figure 2, each approach exhibits distinct advantages and limitations, determining its applicability to specific monitoring environments and operational conditions.

### 3.1. Culture-Based Monitoring Methods

**Figure 3:**
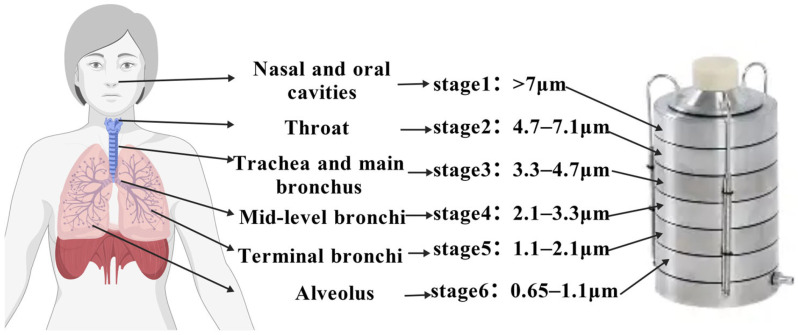
Schematic of Andersen six-stage impactor corresponding to human deposition (created with BioGDP.com [9]).

Culture-based monitoring methods employ artificial culture media to selectively culture microorganisms in bioaerosols, relying on metabolic traits of microorganisms to form visible colonies under specific conditions, thereby enabling qualitative and quantitative assessment of targeted fungal or bacterial concentrations in the air. The Andersen six-stage (or eight-stage) cascade impactor is the most widely used sampler in field measurements. By simulating respiratory tract deposition characteristics (Figure 3), it can directly collect airborne microorganisms ranging in size from 0.65 to 7 μm onto designated Petri dishes. Furthermore, based on different cut-off particle sizes, it facilitates the determination of the size distribution of culturable bioaerosols at the sampling site [6]. For instance, Tsay et al. [85] employed an Andersen six-stage sampler to investigate the influence of patient visits on bacterial distribution within the Intensive Care Unit (ICU). Their findings indicated that during visits, the proportion of bacteria measuring 1.1–4.7 μm was approximately 1–2.4 times greater than that observed prior to visits. Nonetheless, these inherent limitations may reduce microbial viability, and some microorganisms show low recovery rates due to poor adhesion to agar media [86].

### 3.2. Microscope-Based Monitoring Methods

Microscopy-based monitoring methods examine bioaerosol particles directly by means of optical or electron magnification, often combined with staining for qualitative and semi-quantitative analysis, including optical microscopy, fluorescence microscopy, and electron microscopy. Notably, optical microscopy is typically used only for rough counting of bioaerosol particles, leading to comparatively high analytical uncertainty [87]. Fluorescence microscopy operates by detecting either the intrinsic autofluorescence of biological components or the signal from applied fluorescent dyes [88]. The intensity of these fluorescence signals is used to estimate bioaerosol concentration, a feature that renders the method highly effective for low-concentration detection. In addition, electron microscopy identifies viruses by combining ultrastructural morphology observed under the electron beam with biological characteristics of viral nucleic acids and proteins [89]. Specifically, electron microscopy can achieve specific identification of individual bioaerosol particles.

Nevertheless, microscopy-based detection of bioaerosols suffers from notable limitations. It is a highly demanding technique that requires substantial operator expertise, resulting in procedures that are both time-consuming and labor-intensive.

### 3.3. Molecular Biology-Based Monitoring Methods

Molecular biology-based bioaerosol monitoring methods achieve qualitative and quantitative analysis by detecting microbial DNA or RNA in samples. These methods are suitable for identifying all microorganisms present in the air. With advantages such as high sensitivity, strong specificity, and rapid detection, molecular biology techniques are widely used in environmental quality assessment and airborne pathogen monitoring [90,91], which commonly apply methods including Polymerase Chain Reaction (PCR) [92], quantitative PCR (qPCR) [93], Denaturing Gradient Gel Electrophoresis (DGGE) [94], and various sequencing technologies. PCR is a standard method for microbial detection, enabling logarithmic amplification of target DNA regions via specific primers and thermostable DNA polymerase [95]; owing to its specificity, sensitivity, and short processing time, PCR holds great potential for studying biological components in indoor aerosols. Compared with traditional PCR, real-time quantitative PCR (qPCR) offers faster results, lower variability, higher sensitivity, and direct quantifiability [91], as it continuously monitors fluorescence from labeled amplification products during the reaction [96]. While PCR is widely used to identify waterborne and foodborne bacterial and viral pathogens [93], its application is expanding in atmospheric bioaerosol research [97], and it is increasingly being combined with other methods in emerging studies, such as DGGE [98] and Terminal Restriction Fragment Length Polymorphism (T-RFLP) [99], which serve as valuable post-PCR analytical tools [100].

In recent years, high-throughput sequencing has been extensively applied to characterize microbial community diversity and composition in atmospheric environments. This approach extracts and clones total environmental microbial DNA to construct metagenomic libraries. By use of systematic analysis of nucleic acids, the community structure, species diversity, metabolic potential, and functional diversity are provided without the need for cultivation [101,102]. Since most environmental microorganisms are difficult to culture on low-nutrient media, traditional quantification methods often fail to detect them [103,104]. High-throughput sequencing serves as a powerful tool for the comprehensive profiling of all microorganisms present in a sample. Common sequencing platforms include Roche 454 pyrosequencing, Illumina MiSeq, and HiSeq among second-generation technologies. Notably, Illumina MiSeq has emerged as a mainstream platform due to superior accuracy, higher throughput and lower cost [105]. Depending on research objectives and budgets, two main strategies are commonly employed, involving either amplicon sequencing of marker genes for rapid microbial community analysis or whole-genome shotgun sequencing for comprehensive assessment of gene composition and functional potential. In pathogen identification, 16S rRNA amplicon sequencing is widely used for preliminary screening and classification due to its speed and cost-effectiveness, especially for uncultured or hard-to-identify bacteria [105,106]. However, 16S rRNA sequencing typically resolves organisms to the genus or species level. When strain-level identification, genomic structure analysis, or direct functional gene profiling is required, whole-genome shotgun sequencing offers decisive advantages [107].

### 3.4. Immunology-Based Monitoring Methods

Immunoassays detect target bioaerosols by exploiting the specific binding interaction between antibodies and their corresponding antigens on the particle surface [108]. Through enzyme-catalyzed color development, fluorescence signal amplification, or colloidal gold labeling, the binding event is converted into detectable physical signals, thereby enabling qualitative or quantitative analysis. Representative immunoassay techniques include Enzyme-Linked Immunosorbent Assay (ELISA), immunofluorescence staining, immunoblotting, and Lateral Flow Immunoassay (LFIA) [109]. ELISA is extensively employed for detecting allergens in bioaerosols, owing to high sensitivity and quantitative capability, which has become an essential tool for investigating the airborne behavior of allergens against the backdrop of a globally rising allergic population [108]. It not only confirms the presence of target allergens in the air but also delivers accurate data on their concentrations and temporal dynamics [110]. Furthermore, analysis of the obtained concentration gradient information allows for inference of the dispersal range and diffusion patterns of allergens [111].

### 3.5. Monitoring Methods Based on Physicochemical Properties

Bioaerosol particles contain specific organic molecules, such as proteins, coenzymes, cell wall components, and pigments, which fundamentally distinguish them from non-biological aerosols in chemical composition. Methods based on physicochemical properties infer the presence and transmission risks of bioaerosols by analyzing their autofluorescence signatures or single-particle chemical composition. These approaches (e.g., fluorescence spectroscopy, aerodynamic particle sizing, and mass spectrometry) offer notable advantages, including strong real-time capability, suitability for online analysis, and applicability to large-scale screening.

#### 3.5.1. Fluorescence Spectroscopy Technology

Fluorescence spectroscopy detects bioaerosols by using lasers at specific wavelengths to excite intrinsic fluorophores within the particles, thereby inducing characteristic fluorescence. The signals are then collected and recorded by photomultiplier tubes, often presented as spectral or image data [112]. Laser-Induced Fluorescence (LIF) is recognized for enabling non-invasive, in situ, high-time-resolution particle detection [113]. Recent advancements have yielded a range of instruments, including the Wideband Integrated Bioaerosol Sensor (WIBS), Ultraviolet Laser-Induced Fluorescence LiDAR (UV-LIF LiDAR), and Ultraviolet Aerodynamic Particle Sizer (UV-APS).

Notably, parameters of individual aerosol particles can be measured by UV-APS in real time [114]. By virtue of high resolution in detecting viable biological particles, UV-APS is valuable for studying indoor air quality and the effects of atmospheric conditions on particle fluorescence [115,116]. In addition, systems based on LIF principles enable real-time monitoring. For instance, WIBS provides real-time data on particle size, shape, and fluorescence [117,118]. Fluorescence LiDAR systems are considered a key research direction for developing biological defense capabilities in many countries [115]. Furthermore, with advances in artificial intelligence and holographic imaging, real-time particle detectors based on light scattering and fluorescence are continuously evolving. Commercially available instruments include the Rapid-E+ [119], SwisensPoleno Jupiter [120], BioScout [121], MBS [122], SIBS [29], etc. These emerging tools, each with distinct characteristics suitable for different environments, have significantly enhanced the capability for bioaerosol particle identification.

#### 3.5.2. Raman Spectroscopy

Raman spectroscopy identifies bioaerosol particles by analyzing the inelastic scattering of monochromatic laser light, which excites molecular vibrational modes and produces characteristic spectral fingerprints [123,124]. With advantages such as single-particle analysis, rapid signal acquisition, and high specificity [125,126], Raman spectroscopy is increasingly being applied in microbiological studies. However, conventional Raman signals are inherently weak and susceptible to interference [127], leading to potential errors in bioaerosol identification. To address these problems, various signal enhancement techniques have been developed, including Surface-Enhanced Raman Scattering (SERS) [128], Ultraviolet Resonance Raman (UVRR) [126], Tip-Enhanced Raman Scattering (TERS) [129], and Coherent Anti-Stokes Raman Spectroscopy (CARS) [130].

#### 3.5.3. Mass Spectrometry Technology

Mass spectrometry achieves rapid microbial identification by analyzing the mass-to-charge ratios of ionized sample components to obtain characteristic protein or biomolecular mass fingerprints. By comparing these fingerprints against reference spectral databases [131], it enables rapid microbial identification and can support real-time bioaerosol monitoring. Based on the use of an ionization matrix, the technology is broadly divided into two categories. The first encompasses matrix-free laser desorption/ionization methods such as Bioaerosol Mass Spectrometry (BAMS), which allows for real-time, single-particle analysis without reagents or sample preparation [132]. However, it faces limitation including the relatively low ion transmission efficiency, detection limits, and sensitivity [133]. The second is Matrix-Assisted Laser Desorption/Ionization Mass Spectrometry (MALDI-MS), which employs a matrix that co-crystallizes with the analyte, providing a soft ionization method that minimizes molecular fragmentation [134]. By preserving molecular integrity, MALDI-MS achieves high resolution for distinguishing similar particle types and is frequently used to detect environmental pollen, as well as pathogenic or allergenic microorganisms [135].

## 4. Conclusions and Prospects

This review synthesizes the current understanding of the source characteristics, source apportionment, and monitoring technological advancements concerning atmospheric bioaerosols. The community composition of atmospheric bioaerosols is influenced by seasonal and environmental factors, with elevated risks of pathogen and antimicrobial resistance gene dissemination observed in specific habitats such as wastewater treatment plants and landfills. The complexity of pollution sources has made source apportionment techniques, especially those relying on database comparisons and probabilistic models, indispensable for accurate quantitative tracing. Bioaerosol monitoring technology is evolving towards higher sensitivity, throughput, and real-time online capability. While traditional culture-based methods, such as Anderson impactor sampling, remain standard, they suffer from operational complexity, inability to detect non-culturable microorganisms, and potential impacts on microbial viability. Microscopy allows for direct morphological observation but is labor-intensive and time-consuming. In contrast, molecular biology-based methods overcome culture-based methods limitations, enabling comprehensive analysis of microbial community structures and functional genes. Immunology-based methods offer distinct advantages for the quantitative detection of allergens. Techniques based on physicochemical properties facilitate the real-time, rapid identification of single bioaerosol particles, significantly enhancing emergency response capabilities in public health scenarios.

With growing research demands related to bioaerosols in ecological evolution, climate change, human health impacts, and public health safety, higher standards are being placed on the precision, efficiency, and applicability of detection and source-tracing technologies. Considering the current state of development in bioaerosol source apportionment and detection, future efforts in the field should prioritize the following directions:

(1) Systematic Establishment of Characteristic Bioaerosol Databases: Develop comprehensive databases encompassing locally relevant sources, such as specific soil types, dominant plant species, and characteristic agricultural or aquacultural activities.
(2) Coupled Real-time Online Monitoring and Deep Biological Analysis Technologies: Future development will focus on creating portable, automated integrated systems, which enable high-frequency early-warning screening via online spectroscopic technologies while incorporating molecular biology modules for precise identification, thereby meeting the demands for rapid response in public health emergencies.
(3) Optimize the application of source apportionment models and artificial intelligence algorithms based on complex environments. Further explore the application of machine learning and AI algorithms in bioaerosol source tracing. By integrating these with established source apportionment methods, the goal is to establish high-precision models capable of dynamically quantifying the contribution rates of multiple pollution sources.

## Figures and Tables

**Table 1:** Bioaerosol emission characteristics of typical man-made systems.

Sources of Samples	Location	Methods	Major Characteristic	References
WWTP	China	Culture-based methods	Culturable *Staphylococcus aureus* Concentration: 0.43–134.45 CFU/m^3^	[24]
WWTP	China	16S rRNA gene sequencing and culture-based methods	Culturable Bacterial Concentration: 21.2–1.43 × 10^3^ CFU/m^3^	[25]
WWTP	China	SEM-EDS, llumina gene sequencing and culture-based methods	Bacteria in aerosols are mostly attached to mineral particles containing silicon (Si), calcium (Ca) and iron (Fe)	[26]
WWTP	Polska	In situ hybridization (FISH) and cultivation methods	The FISH method gave values up to 10^3^-fold times greater than those obtained by the cultivation method	[27]
WWTP	Finland	Enzyme-Linked Immunosorbent Assay (ELISA)	Colonies of *Ochrobactrum anthropi*, *Pantoea agglomerans* and *Stenotrophomonas maltophilia* were isolated from the workplaces	[28]
Landfill	UK	Spectral Intensity Bioaerosol Sensor (SIBS)	The average number of concentrations of total particles (NT) and fluorescence particles (NF) were both higher at WWTPs (NT = 2.01 cm^−3^, NF = 1.13 cm^−3^) than the background site (NT = 1.79 cm^−3^, NF = 1.01 cm^−3^)	[29]
Landfill	China	Illumina sequencing and culture-based methods	Culturable Bacterial Concentration (Workplace): (3179 ± 299)–(8051 ± 919) CFU/m^3^ Culturable Bacterial Concentration (Cover Area): (931 ± 124)–(3835 ± 385) CFU/m^3^	[30]
Landfill	UK	Scanning electron microscopy and cultivation methods	Most aggregates consisted of clusters of 2–3 particles as opposed to chains and were <10 μm in size	[31]
Landfill	Korea	*Limulus* amoebocyte lysate (LAL)	The average exposure level to total dust was 0.9 mg/m^3^ (range = 0.05 to 4.51 mg/m^3^), and the average exposure to endotoxin was 1123 EU/m^3^	[32]
Landfill	Ireland	Waveband Integrated Bioaerosol Sensor model 4 (WIBS-4)	0.5–3 μm with morphologies ranging from elongated to ellipsoidal/spherical	[33]
Livestock farm	Austria	16S rRNA gene sequencing and culture-based methods	Culturable Mesophilic Bacterial Concentration: 6.2 × 10^5^ CFU/m^3^ Culturable *Staphylococcus* Concentration: 8.8 × 10^4^ CFU/m^3^	[34]
Livestock farm	America	Kinetic *Limulus* amebocyte lysate (LAL) assay and the rFC assay	Ratios of LAL to rFC were 0.89 for air samples from the field study and 1.06 for the laboratory-generated air samples	[35]
Livestock farm	Denmark	DAPI staining combined with quantitative FISH microscopic observation	Firmicutes as the dominant group with *Streptococcus* as the major genus, while *Actinobacteria* constituted 10% of the detectable bacteria	[36]
Livestock farm	Australia	The ultraviolet aerodynamic particle sizer (UVAPS) and culture-based methods	The concentrations of both viable (fluorescent) and total (fluorescent and non-fluorescent) particles inside the swine confinement building were in order of 10^6^–10^7^ particles m^−3^	[37]
Hospital	China	16S rRNA gene sequencing and culture-based methods	Total Culturable Bacteria (Mean ± SD): 193 ± 39 CFU/m^3^. Culturable Bacterial Concentration (Hospital Wards): 205 ± 40 CFU/m^3^ Culturable Bacterial Concentration (Outpatient Waiting Area): 168 ± 31 CFU/m^3^	[38]
Hospital	America	Ultraviolet Aerodynamic Particle Sizer (UV-APS)	The detection efficiency of the UV-APS was ≥99% for all particle generation rates and species	[39]
Hospital	Korea	Kinetic *Limulus* amebocyte lysate (LAL) assay and culture-based methods	Average total airborne endotoxin levels ranged from <0.01 to 10.02 EU m^−3^, with an overall mean of 1.03 EU m^−3^	[40]
Hospital	India	Microscopic examination	In the blood agar plates, the *S. epidermidis* was found to be a maximum 62%, micrococcus was 22%, diphtheroid was 10%, fungi 4% and the *S. aureus* was lowest at 2%	[41]

## Data Availability

No new data were created or analyzed in this study. Data sharing is not applicable to this article.
